# Coronary Computed Tomography Angiography in Combination with Coronary Artery Calcium Scoring for the Preoperative Cardiac Evaluation of Liver Transplant Recipients

**DOI:** 10.1155/2017/4081525

**Published:** 2017-01-10

**Authors:** Jae Moon Choi, Yu-Gyeong Kong, Joon-Won Kang, Young-Kug Kim

**Affiliations:** ^1^Department of Anesthesiology and Pain Medicine, Asan Medical Center, University of Ulsan College of Medicine, Seoul, Republic of Korea; ^2^Department of Radiology, Asan Medical Center, University of Ulsan College of Medicine, Seoul, Republic of Korea

## Abstract

Liver transplantation is the best treatment option for early-stage hepatocellular carcinoma, liver cirrhosis, fulminant liver failure, and end-stage liver diseases. Even though advances in surgical techniques and perioperative care have improved postoperative outcomes, perioperative cardiovascular complications are a leading cause of postoperative morbidity and mortality following liver transplantation. Ischemic coronary artery disease (CAD) and cardiomyopathy are the most common cardiovascular diseases and could be negative predictors of postoperative outcomes in liver transplant recipients. Therefore, comprehensive cardiovascular evaluations are required to assess perioperative risks and prevent concomitant cardiovascular complications that would preclude good outcomes in liver transplant recipients. The two major types of cardiac computed tomography are the coronary artery calcium score (CACS) and coronary computed tomography angiography (CCTA). CCTA in combination with the CACS is a validated noninvasive alternative to coronary angiography for diagnosing and grading the severity of CAD. A CACS > 400 is associated with significant CAD and a known important predictor of posttransplant cardiovascular complications in liver transplant recipients. In this review article, we discuss the usefulness, advantages, and disadvantages of CCTA combined with CACS as a noninvasive diagnostic tool for preoperative cardiac evaluation and for maximizing the perioperative outcomes of liver transplant recipients.

## 1. Introduction

Since the first successful liver transplantation was reported in 1963 [1], this procedure has been performed to treat hepatocellular carcinoma at early stages, liver cirrhosis, fulminant liver failure, and end-stage liver diseases. Advances in surgical techniques, organ preservation, and perioperative care including immunosuppression have further improved the perioperative outcomes of liver transplantation [2]. As the average age of patients undergoing liver transplantation continues to increase, perioperative cardiovascular complications are a leading cause of morbidity and mortality after liver transplantation [3]. Previous cardiac disease, adverse intraoperative cardiovascular events, and an integrated model for end-stage liver disease score are known as independent predictors of cardiovascular complications for the 6-month period after liver transplantation [4].

The American College of Cardiology/American Heart Association guidelines recommend cardiovascular evaluation for individuals undergoing noncardiac surgery [5]. As with any patient being considered for a surgical procedure, individuals with end-stage liver disease should have an evaluation for cardiac function and coronary heart disease. Since the incidence of perioperative cardiovascular complications varies from 25% to 70% in liver transplant recipients [3, 6, 7], these patients need to be very thoroughly evaluated for cardiac function. Ischemic coronary artery disease (CAD) and cardiomyopathy are the most common cardiovascular diseases and could be negative predictors of postoperative outcomes in liver transplant recipients [8]. The prevalence of CAD ranges between 2.5% and 12% in patients undergoing orthotopic liver transplantation [9–12]. The history of CAD is also reported to be an important risk factor for postoperative acute coronary syndrome in liver transplant recipients [13]. Taken together, the evidence to date indicates that meticulous cardiovascular evaluations are required to assess perioperative risks and to prevent concomitant cardiovascular complications that would preclude good outcomes in patients undergoing liver transplantation.

A scientific statement from the American Heart Association and the American College of Cardiology Foundation gives a class I recommendation to screen all potential liver transplant candidates for cardiovascular disease initially with a history and physical examination [14]. Noninvasive stress echocardiography is needed as an initial screening test in liver transplant candidates, to assess the cardiac risk [15]. Pretransplant cardiac revascularization is recommended in liver transplant candidates with significant coronary artery stenosis [15]. Newer and more sophisticated imaging modalities, such as cardiac computed tomography and cardiac magnetic resonance imaging, have allowed for more precise diagnostic cardiovascular testing [5]. The two major types of cardiac computed tomography are the coronary artery calcium score (CACS) and coronary computed tomography angiography (CCTA). CCTA with contrast allows for imaging of the heart chambers, coronary arteries, and pulmonary vessels in three dimensions. CCTA was introduced as a noninvasive diagnostic method for evaluating CAD and improving postoperative outcomes by detecting obstructive coronary plaques that result in luminal diameter narrowing in one or more coronary arteries and can be used in combination with CACS. In CACS, pictures are taken of the heart to investigate the calcium deposits in the coronary arteries. Coronary artery calcium deposits are only present in atherosclerotic arteries [16] and represent a very specific sign of CAD. Increases in the calcium deposits in coronary arteries increase the risk of a heart attack or other cardiovascular complications [17].

CCTA has a negative predictive value of 97–99% for predicting the absence of obstructive CAD [18, 19]. In addition, a CACS > 400 on CCTA is known to be predictive of cardiovascular complications within 1 month of a liver transplantation [20]. We here review the usefulness, advantages, and disadvantages of CCTA combined with CACS as a noninvasive diagnostic tool for evaluating preoperative CAD and for reducing postoperative cardiac complications in liver transplant recipients.

## 2. Preoperative Cardiac Evaluation Tests

Preoperative testing for cardiovascular evaluation is highly recommended in liver transplant candidates with a history of cardiovascular disease, alcoholism, or diabetes mellitus, especially in patients > 60 years of age [21]. However, cardiovascular evaluations are challenging in liver transplant candidates. The majority of these patients cannot undergo cardiopulmonary exercise testing due to deconditioning, malnutrition-associated muscle weakness, ascites, anemia, and cirrhotic cardiomyopathy [22]. Preoperative cardiac assessments in patients undergoing liver transplantation include electrocardiography, cardiopulmonary exercise testing, basal and dobutamine stress echocardiography, myocardial perfusion imaging by single-photon emission computed tomography (SPECT), coronary angiography, and cardiac computed tomography [23].

### 2.1. Electrocardiography

Preoperative resting electrocardiography is a noninvasive test used to obtain diagnostic and prognostic information on liver transplant recipients [5]. Standard 12-lead electrocardiography is useful for continuously recording pulse generation, heart rhythm, conduction disturbances, and ischemic changes. One of the most important electrocardiographic parameters in patients with liver cirrhosis is the prolongation of the QT interval [27]. A corrected QT interval > 450 ms indicates an increased risk of ventricular arrhythmia and sudden cardiac death [28]. However, it is helpful to perform pharmacological stress testing, such as dobutamine stress echocardiography and stress SPECT, to assess functional capacity in liver transplant recipients [5].

### 2.2. Cardiopulmonary Exercise Testing

Preoperative cardiopulmonary exercise testing is a safe, noninvasive method to determine the cardiopulmonary reserve in liver transplant recipients. The preoperative cardiopulmonary reserve assessed by submaximal cardiopulmonary exercise testing represents a sensitive and specific predictor of early survival after liver transplantation [29]. In patients undergoing liver transplantation, impaired anaerobic threshold is related to postoperative hospitalization, survival, and mortality [30]. Because most liver transplant recipients are too debilitated to complete cardiopulmonary exercise testing, many centers conduct pharmacological stress test using dipyridamole, dobutamine, or adenosine [14].

### 2.3. Dobutamine Stress Echocardiography

Dobutamine stress echocardiography has been introduced as an initial screening test for coronary heart disease. The American Association for the Study of Liver Disease recommends dobutamine stress echocardiography as an effective screening tool for evaluating CAD in patients undergoing liver transplantation [31]. Currently, the American College of Cardiology and the American Heart Association recommend that noninvasive stress testing may be considered for liver transplant candidates who have ≥ 3 risk factors for CAD [5]. A previous meta-analysis has suggested that dobutamine stress echocardiography detects CAD with a high degree of sensitivity and specificity in the general population [32]. Although dobutamine stress echocardiography is commonly used to evaluate risk stratification, it does not accurately reflect the severity of obstructive CAD in liver transplant candidates [33, 34]. In a subset analysis of orthotopic liver transplant candidates, dobutamine stress echocardiography compared with coronary angiography has a 75% sensitivity and 57% specificity in detecting CAD [12]. Dobutamine stress echocardiography has a 9% sensitivity, 33% positive predictive value, and 89% negative predictive value for predicting early cardiac events after liver transplantation [13].

The use of *β*-blocking agents for the prevention of esophageal variceal bleeding in end-stage liver disease has been found to be a common cause of failure to achieve the target heart rate in dobutamine stress echocardiography. The previously reported results of dobutamine stress echocardiography were inconclusive in 19–21% of patients on *β*-blocking agents [35, 36]. In addition, when *β*-blocking agents are stopped to enable dobutamine stress echocardiography, there is an increased risk of variceal bleeding [37].

### 2.4. SPECT

SPECT is the most widely known nuclear test for evaluating myocardial perfusion using diffusible radiotracers. The sensitivity of SPECT is approximately 90%, and the specificity is 75–80% in pharmacological stress studies that use thallium [38]. In liver transplant candidates, however, SPECT imaging is known to be an inaccurate screening test. The sensitivity of SPECT is 37% and its positive predictive value is 22% in comparison with coronary angiography in liver transplant candidates [39]. Adenosine-SPECT has a sensitivity of 62% and a positive predictive value of 30% for diagnosing severe CAD in patients with end-stage liver disease [40]. In patients undergoing orthotopic liver transplantation, SPECT has a sensitivity of 57%, a positive predictive value of 28%, and a negative predictive value of 91% for predicting early cardiac events [13]. A primary deficiency of SPECT is associated with the vasodilating agents used (adenosine and dipyridamole). Chronically decreased arterial vascular resistance in patients with advanced liver failure may limit the typical vasodilating response of the coronary arteries to adenosine or regadenoson [39, 40].

### 2.5. Coronary Angiography

The current standard for the diagnosis of symptomatic obstructive CAD is coronary angiography [41]. Coronary angiography is known as a superior diagnostic tool for evaluating coronary heart disease. The main advantage of coronary angiography is that it can be diagnosed and treated simultaneously with immediate percutaneous coronary intervention. With the lack of high-level evidence for the superiority of noninvasive tests, many centers rely on invasive coronary angiography [42]. Significant CAD is defined as a more than 50% decrease in the lumen diameter resulting in a hemodynamically significant reduction in coronary blood flow [43]. The increased use of coronary angiography and percutaneous coronary intervention before orthotopic liver transplant is also associated with significant reductions in postoperative coronary events and all-cause mortality [44]. In contrast, coronary interventions do not reduce mortality rates in orthotopic liver transplant patients with severe CAD [45]. The authors of that study proposed that patients who undergo coronary intervention prior to liver transplantation are at high risk of death from a cardiac event.

Coronary angiography in patients with relatively advanced liver disease is more likely to increase the risk of vascular complications, such as bleeding, due to coagulation abnormalities secondary to thrombocytopenia and prolonged prothrombin time [46]. According to recent studies, transradial cardiac catheterization appears to be a safe method in liver transplant candidates despite significantly lower platelet count and higher international normalized ratio [47, 48]. No adverse events were recorded after coronary angiography in 84 orthotopic liver transplant candidates [49]. However, it remains unclear when to proceed with invasive coronary angiography. A standardized protocol for assessing CAD in liver transplant recipients is therefore needed.

## 3. CCTA

Noninvasive coronary imaging has been a topic of great research interest for a number of years [50]. CCTA is validated as a potential alternative to coronary angiography for diagnosing and grading the severity of CAD in a large number of patients [19, 51]. The main obstacles to interrupting the noninvasive visualization of coronary arteries include cardiac motion, small vessel size, and the need for elevated intravascular contrast resolution [52]. The advent of multidetector computed tomography has enabled the acquisition of excellent anatomic details of the coronary arteries in a beating heart. Multidetector computed tomography has the potential to considerably reduce the radiation dose and the amount of contrast agent required while maintaining high diagnostic accuracy [53, 54]. Multidetector computed tomography also has the capability to simultaneously and continuously obtain multiple images. Approximately 300 transaxial images with a thickness of 0.5–1 mm are obtained during a single breath-hold. Through the use of electrocardiographic data, multidetector computed tomography images can be reconstructed at the optimal cardiac phases that have no or minimal coronary artery motion.

The findings obtained from coronary angiography are limited to information regarding the coronary artery lumen and cannot identify the accumulation of atherosclerotic plaques in the coronary vessel wall. However, CCTA can delineate the coronary anatomy in three dimensions and noninvasively visualize coronary vessels in any desired spatial orientation using the acquisition of volumetric data sets. Manipulation of the images through prospectively electrocardiogram-triggered high-pitch spiral acquisition offers distinct advantages in comparison with coronary angiography [55].

An atherosclerotic lesion is defined by intimal and smooth muscle cell proliferation, lipid accumulation, and connective tissue deposition [56]. Atherosclerosis eventually causes the obstruction of blood flow and leads to clinical symptoms. CCTA acquires detailed images of calcified and noncalcified plaques [57]. CCTA has the potential to detect the length, morphology, and composition of atherosclerotic plaques in stenotic regions [58–61]. More research is needed to compare atherosclerotic plaque characteristics such as site, length, composition, and morphology between liver transplant candidates and other populations.

Clinically significant but not critical coronary artery stenosis on CCTA is defined as the narrowing of the coronary artery diameter by 50% to 70% [62, 63]. There is debate about whether CCTA should be considered for patients with end-stage liver disease [21]. Routine preoperative CCTA has a low yield in patients evaluated for liver transplant. In a previous study of 1045 cirrhotic patients with no history of chest pain or CAD, CCTA revealed a similar frequency of obstructive CAD in the cirrhotic (7.9%) and healthy (7.2%) cohorts [64]. Twenty-four of the patients in that study with obstructive CAD with CCTA were referred for cardiac catheterization, and only 6 ultimately underwent revascularization [64]. In several previous meta-analyses, however, multidetector computed tomography has demonstrated a 98-99% sensitivity and 89–91% specificity for the detection of coronary plaques [65–67]. CCTA has also shown a good negative predictive value (83–99%) for excluding significant CAD [68]. A normal scanning result in CCTA can effectively exclude obstructive CAD and abolish the need for further investigation [21]. CCTA combined with regadenoson-induced stress computed tomography perfusion is a stress test with a high diagnostic performance in assessing intermediate coronary artery stenosis in asymptomatic patients [69]. However, there have been no previous reports that compared CCTA and invasive coronary angiography for detecting CAD in liver transplant recipients. Further studies are thus needed to determine the diagnostic accuracy of CCTA in comparison with coronary angiography for the detection of coronary artery stenosis and in making interventional decisions in liver transplant candidates.

## 4. CACS

CACS—as estimated by noncontrast, electrocardiography-gated computed tomography—is an established noninvasive tool for the identification and quantification of calcified plaques in a coronary artery [70]. Calcium phosphate and hydroxyapatite are responsible for the calcification of the coronary artery. Coronary artery calcium deposits can be measured rapidly and noninvasively using computed tomography. The presence of calcium in a coronary artery is defined by the presence of any pixel within the region of interest with a computed tomography density > 130 Hounsfield units due to noise [70, 71]. A density factor derived from the peak brightness of each calcium focus and its area on a computed tomography scan have been used to determine the calcium score for each scan using the method developed by Agatston et al. [70]. The calcium scores for each lesion were then summed to define the total CACS for each patient. The quantification of coronary artery calcium on computed tomography is correlated with the severity of luminal narrowing, stenosis severity, and total plaque burden in the artery due to atherosclerotic disease [72].

**Figure 1:**
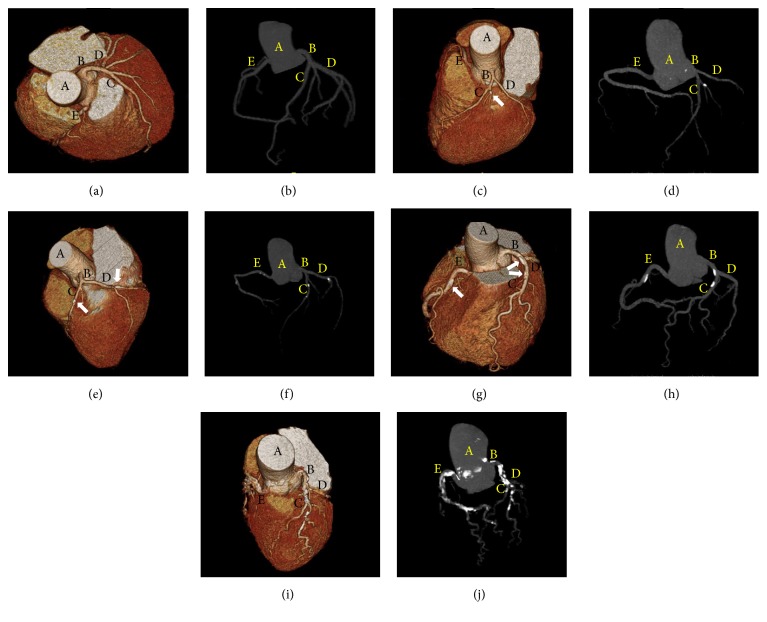
Three-dimensional volume-rendered images (a, c, e, g, and i) and angiographic images (b, d, f, h, and j) of the coronary artery obtained using computed tomographic angiography in patients undergoing liver transplantation. ((a) and (b)) CACS = 0 (absent); ((c) and (d)) CACS = 9 (minimal); ((e) and (f)) CACS = 95 (mild); ((g) and (h)) CACS = 279 (moderate); ((i) and (j)) CACS = 5210 (extensive). Arrows indicate coronary calcified plaques. A, aorta; B, left main coronary artery; C, left anterior descending artery; D, left circumflex artery; E, right coronary artery. CACS, coronary artery calcium score.

CACS values are generally classified as absent (0), minimal (1–10), mild (11–100), moderate (101–400), or extensive (>400) (Figure 1) [73]. A CACS < 10 indicates the absence of any significant coronary obstructive lesion. CACS is an independent predictor of coronary heart disease risk and mortality and reflects the prevalence and extent of atherosclerosis. In several meta-analyses undertaken to date, a higher CACS has been associated with a greater degree of coronary artery stenosis and a higher risk of coronary heart disease [74–76]. In the general population, a doubling of the CACS increases the probability of coronary events by 25% during a median follow-up period of 3.8 years [77]. In a previous large prospective study that followed up 44,052 patients over a 5-year period, the mortality rate associated with a CACS ranging from 1 to 10 was 1.06%, which was higher than in cases with a CACS of 0 (0.52%) and lower than in patients with CACS > 10 (3.96%) [78]. A CACS > 400 is significantly associated with the presence of coronary artery stenosis on coronary angiography in asymptomatic patients and liver transplant candidates [25, 73].

CCTA combined with CACS is well tolerated in comparison with the stress test and is a useful noninvasive technique for assessing CAD in patients with end-stage liver disease. The prognostic value of CCTA is comparable to that of dobutamine stress echocardiography and it has a negative predictive value of 95% for major adverse cardiac events in the 1-year posttransplant follow-up period in orthotopic liver transplant recipients [10]. Kong et al. reported a mean CACS on CCTA of 42 ± 195 in 443 liver transplant candidates and that 11 (2.5%) patients were categorized into the extensive groups [20]. Based on that study, a CACS > 400 is an important predictor of early cardiovascular complications such as a nonfatal myocardial infarction, serious arrhythmia, and cardiac death after liver transplantation [20]. Increasing age, male sex, and diabetes mellitus have also been associated with a CACS > 400 in liver transplant recipients [79]. CCTA is recommended for preoperative cardiovascular assessment in liver transplant candidates who had a diagnosis of diabetes mellitus or ≥ 2 traditional risk factors for CAD (age > 45 years for male or > 55 years for female, hypercholesterolemia, hypertension, tobacco use, and family history of early CAD) [80]. Therefore, we suggest CCTA combined with CACS for preoperative cardiac evaluation in liver transplant recipients with the above-mentioned CAD risk. Coronary angiography is likely to be performed in patients with coronary artery stenosis ≥ 50% on CCTA or CACS > 400.

However, there is still limited information on the predictive ability of the CACS with respect to perioperative outcomes in patients undergoing liver transplantation (Table 1). More studies are needed to clarify this. Furthermore, it is important to understand that CACS should be used to indicate coronary angiography with possible interventional procedures to reduce the risk for perioperative acute cardiac events.

## 5. Advantages and Disadvantages of CCTA Combined with CACS

Noninvasive CCTA reduces the need for invasive coronary angiography and can be safely used in the perioperative cardiovascular risk assessment of liver transplant candidates during the posttransplant follow-up period [24]. CACS is significantly associated with cardiovascular risk factors, such as age and the involved number of coronary vessels, and is a more sensitive detector of cardiovascular risk factors than the Framingham risk score in liver transplant recipients [81]. Detecting an increase in coronary artery calcium has the potential to identify a risk for increased CAD across all age groups [82]. CACS is also a useful tool for risk stratification in both younger and elderly patients. In addition, the coronary artery calcium area has been shown to be reflective of the atherosclerotic plaque burden within the coronary system [71]. Serial evaluations of the CACS by computed tomography provide information regarding the progression, stabilization, and regression of coronary artery atherosclerosis. Coronary angiography has high interobserver and intraobserver variability during interpretation [41]. However, the interobserver and intraobserver variability in CACS on computed tomography has been shown to be excellent [83, 84].

The necessary radiation dose for CCTA has been found previously to be in the range of 8–21 mSv, which is higher than that associated with conventional coronary angiography (2–5 mSv) [85]. However, improvements in computed tomography technology and software quality have allowed significant decreases in radiation doses for CCTA image acquisition below 1 mSv [55, 86]. A given CACS must be compared with the score of an average person matched for sex, age, and risk factor profiles for coronary heart disease [87]. The same CACS may have different implications in different people depending on their sex, age, and risk factor profiles. Related factors, including heart rate and irregular heart rhythm, can interfere with the diagnostic quality of the images [88]. Ascites, dyspnea, orthopnea, and altered mental status caused by hepatic encephalopathy can affect breath-holding ability in liver transplant recipients. In addition, the coronary artery lumen on CCTA can be obscured in a region of severe coronary calcification or in the presence of a coronary stent. CCTA also presents difficulties when assessing distal coronary artery segments and some side branches with a diameter < 1.5 mm [89].

## 6. Conclusion

Notwithstanding diverse clinical experiences and various advances in knowledge, no gold standard has yet been developed for cardiac evaluation in liver transplant candidates. Due to an equal or high incidence of CAD associated with significant morbidity in these patients, the development of a screening protocol with a reliable predictive value is still required. Given that the clinical applications that can be used in liver transplant candidates remain limited, CCTA combined with CACS seems to be a reliable screening option for preoperative noninvasive evaluation of CAD in liver transplant recipients with diabetes mellitus or ≥ 2 traditional risk factors for CAD. Coronary angiography can be performed in liver transplant recipients with coronary artery stenosis ≥ 50% on CCTA or CACS > 400. In addition, CCTA combined with CACS provides useful information for predicting posttransplant cardiovascular complications in patients undergoing liver transplantation.

## Figures and Tables

**Table 1:** Clinical applications of CCTA in combination with the CACS in LT candidates.

Study	Patients (*n*)	Positive criteria; positive patients, *n* (%)	Clinical outcomes
Jodocy et al. [24]	54	CACS > 300 or > 50% stenosis on CCTA; 24 (44%)	CCTA and CACS are useful tools for perioperative cardiovascular risk assessments.
Cassagneau et al. [10]	52	> 50% stenosis on CCTA; 6 (12%)	The prognostic value of CCTA is comparable to dobutamine stress echocardiography.
Chae et al. [11]	247	Mild to moderate involvement on CCTA; 27 (11%)	CCTA should be included in routine pretransplant cardiac workups.
Kemmer et al. [25]	85	CACS > 100; 30 (35%)	CACS is a valid alternative tool for risk stratification of LT candidates.
Kong et al. [20]	443	CACS > 400; 11 (3%)	CACS > 400 is a predictor of cardiovascular complications following LT.
Poulin et al. [26]	100	≥ 70% stenosis on CCTA and/or CAG; 20 (20%)	Using CCTA in the evaluation of LT candidates is challenging but is feasible and safe.

CACS, coronary artery calcium score; CAD, coronary artery disease; CAG, coronary angiography; CCTA, coronary computed tomography angiography; LT, liver transplantation.
